# The Journal

**Published:** 1847-02

**Authors:** 


### PART V.—ABSTRACT.

### ARTICLE XIX.

### THE JOURNAL.

With this number we close the first volume of our enlarged Journal, and the third since its commencement, and having labored hard to render it worthy of the patronage of the profession of the great North-West, we turn our attention to the past, to see how far we have been able to accomplish the designs of our work—which are, to keep our readers advised of what is transpiring of importance in the profession, both at home and in foreign countries; and to afford to the physicians of the North-West, a medium of communication by which the valuable in their observations and reflections may be given to the public, while at the same time, it is made a matter of permanent record.

The Journal is now permanently established, and to those friends who have assisted it by their patronage, and by contributions to its pages, we tender our most hearty thanks; while we solicit from them, for the future, a continuance of their favors,—and especially do we ask that our list, both of contributors and subscribers, may be extended.

That we may at one glance see what has been done the past year, in the way of original communications, we will make a brief abstract of them by way of a recapitulation.

No I. Art. 1.—*Epidemic Erysipilas.* By Prof. Fitch.—This is a disease that has prevailed to a great extent in many locations in the west within the last few years, and perhaps in none more extensively than in the country around it, and in the town of Logansport, Ind., the residence of Dr. Fitch. The article shows that he was not an inattentive observer of the disease and the effects of treatment. He took notes of 213 cases, in 182 of which the attack was in the fauces, tonsils, sterno mastoid muscle, or cervical glands, showing a uniformity truly remarkable, and from which peculiarity in the disease, no doubt, it was for a time known by the appellation of "black tongue," although this symptom was actually present in two cases *only.*

The conclusions of Dr. Fitch are that the disease is decidedly contagious, yet often occurring in consequence of epidemic influences.

The principal reliance in treatment is placed in bleeding, which was very successful when resorted to at an early stage of the disease. Next in importance, in the commencement of the disease, are emetics. The use of cathartics was found important in many cases, of which calomel and castor oil were chiefly relied upon.

A gargle of dilute tinc. of iodine was found to be most efficient as an application to the fauces, and the local application most approved for arresting the spread of the inflammation was blistering in advance of it. For reducing the swelling and violence of the local disease, a lotion of dilute tinc. iodine. Where this failed, free incisions of the skin, or punctures with the lancet, were made to allow the escape of the serum beneath it, with decided advantage.

When the disease attacks pregnant or laying-in women it is almost always fatal, yet Dr. F. cured quite a number of such. He regards it as identical with epidemic puerperal peritonitis.

ART. 2.—*Wahoo.*—(*Euonymus Atropurpureus.*) By E. ANDREW, M.D., now of Peoria.—The bark of the root is the part used. This remedy is said to have been used as a cure for intermittent fever by the aboriginies of this country. However, the strongest recommendation in favor of this, to the profession, new remedy, is in its effects as a hyrdrogogue cathartic, which are produced at the same time that a decidedly tonic influence is exerted upon the system. This renders it appropriate in dropsies attended with general debility; while the peculiar property of not losing its effects by continued use, would seem to point it out, as extremely appropriate in obstinate cases.

As this is a native western medicine, the shrub growing in almost all parts of the country, it becomes the duty of the profession to more extensively investigate its merits, and if it shall prove to be as valuable as Dr. A. regards it, it should be in general use.

Its virtues, in cases of anasarca and ascitis, following long continued intermittent fever, have been pretty thoroughly tested during the last few months in the dispensary of the Rush Medical College, and with the most favorable results. We shall be glad to hear how it succeeds in other hands.

The article is well written, and is on a very interesting subject.

No. II. Art. 1.—*Delirium Tremens.* By E. R. Long, M.D., U. S. Army.—This gives a concise view of the most prevalent opinions in regard to the pathology of the disease, and comes to the conclusion, that as a manifestation of "peculiar morbid excitement of the nervous system" is all the constant condition of the disease, we must refer it to the nervous system, and not to gastritis, meningitis, or any other local affection that is sometimes present and sometimes absent.

But these complications, or attendants, must be respected in treatment—and as gastritis is by far the most common, it will often be found necessary to be exceedingly careful of what is introduced into the stomach.

But independent of complications the anodyne treatment is clearly indicated.

Art. 2.—*Amputations in Scrofulous Diseases.* By Prof. Brainard.—This is an interesting and instructive article, showing by several cases reported from the author's own experience, and from Brodie, Druit, and Tamplin, that in many cases of scrofulous disease, affecting the joints, the limb may be saved by proper and patient application of the appropriate treatment; and that, even after exterior suppuration, caries of the bone, and hectic fever have supervened. Nor is this all—it is shown that in these cases amputation is extremely hazardous, as the scrofulous affection is liable to locate upon the lungs, mesentery, or other vital organs, and thus result in the loss of life.

It should always be remembered that it is a greater triumph in surgery, to save a useful member than to display skill in the performance of an operation.

Art. 3.—*Foreign Bodies in the Organs and Tissues.* By Prof. Herrick.—This article shows that foreign substances lodged in the organs and tissues of the body may give rise to symptoms, which for want of a correct history of the case, may perplex the practitioner. Cases are reported illustrating this fact, in all of which the removal of the substance resulted in a speedy recovery.

Art. 4.—*Case of the Bite of a Mad Dog and its Treatment.* By Dr. Stahl, M.D.—This is an interesting case, from the positive and satisfactory evidence of the rabid condition of the dog, and the wound being on the hand, and not through his clothing; showing a strong probability of the patient being infected but for the treatment. This consisted in the use of the actual cautery to the wounds made by the teeth of the dog, kept running by ungent. cantharid, and ung. Hyd. Precip. rub. cum sulph. cupri., and the administration of ½ grain doses of ext. belladonna, repeated so as to induce the specific effects of the medicine. The patient showed no symptoms of hydrophobia.

No. III. Art. 1.—*Sulph. Quinine in the congestive modifications of Scarlet Fever and Measles.* By D. Stahl, M.D., of Quincy, Ill.—There is no doubt of the efficacy of quinine in the cure of the fevers so prevalent in the West, known by the appellation of "congestive." The author regards this type, which often modifies pneumonia, pleuritis, hepatitis, the exanthemata, and most other diseases of the country, as an adynamic condition of the system dependent upon a want of energy in the nerves, which quinine restores. This condition is very common in scarletina and rubeola, in which the author obtained the happiest results from the free administration of quinine in several cases, the reports of which are interesting to the practitioner.

Art. 2.—*The remote and proximate cause of Bilious Fevers of the South and West, considered in the light of Liebig's Theory of Animal Heat.* By S. G. Armor, M.D., of Rockford.—The author in this article makes many exceedingly interesting suggestions in reference to the influence of diet of a highly carbonized character over the system, and suggests that the abundance of carbon in the blood may be the cause of our fevers, and that the curative action of quinine may be, in effecting a chemical change, in the relative proportion of the constituents of the circulating mass.

It is an interesting inquiry, and if it would lead to a determination of the extent to which certain classes of food are appropriate, to be regulated by the thermometer, we might, but for the sudden and extreme changes in the temperature of the weather interfering, eat food with a certainty of its fitness.

Art. 3.—*Amputation of the Superior Maxillary, Malar and Palate Bones for disease of the Antrum. Recovery.* By Prof. Brainard.—This is an account of one of the most important operations that occur in the practice of the surgeon. The disease for which it was performed was a rapidly growing tumor of a malignant character. It is interesting as showing the extent to which the bones of the face may be removed with safety to life.

Art. 4.—*Case of Mental Excitement allayed by Music.* By Prof. Herrick.—This was undoubtedly a case of incipient insanity, produced by over excitement of the brain while the system was too much prostrated to endure it, and shows the power of what may be termed *moral treatment.* The music succeeded in allaying the excitement, no doubt, by fixing the attention; and by its peculiarly agreeable influence induced sleep after the failure of the most powerful narcotics.

No. IV. Art. 1.—*Observations on Insanity and its Treatment in private practice.* By Prof. Evans.—This is a long article and contains some interesting statistics in reference to the cause of the disease—some appropriate reflections in reference to its nature—and gives a moral and physical treatment to be pursued by practitioners in the country, founded upon the improvements that have been recently made in the management of the insane.

Art. 2.—*Case of Tubucular Deposite in the Cerebellum. Autopsical Examination.* By P. A. Allaire, M.D., of Aurora.—This is a concise and well written account of an interesting case. It shows the importance of autopsical examinations in determining the true nature of disease, and affords additional evidence that M. Gendrin and M. Lévellé are mistaken in asserting that "all cerebral tubercles are encysted."

Art. 3.—*Operation on the closure of the external orifice of the Vaginal Canal.* By A. E. Ames, M.D., of Roscoe.—Atresia of the vagina is of rare occurrence from membranous expansion, and where it does occur it is generally referable to an unnatural development in connexion with, if not of the hymen itself. This case was appropriately and successfully treated at an early age—certainly a much better course than to have left it to obstruct the menstrual flow in after life.

No. V. Art. 1.—*Blindness caused by the use of Sulph. Quinine.* By Prof. McLean.—This article comes in most appropriately at this time, when the members of the profession seem to vie in the freedom with which they use quinine and the magnitude of its doses. Several cases from the author's own experience are reported, showing the effect indicated in the title of the article. As this would be an appropriate time in the experience of practitioners to collect the deleterious effect of this medicine, we hope our friends will not neglect it. Its influence appears to be principally over the nervous system. This is manifested, we think, clearly by its influence in affecting the organs of sense and the intellect. We saw a case of acute mania which lasted about thirty-six hours, in November last, in company with Dr. Brinckerhoff, of Chicago, which had evidently been produced by the patient's taking a 20 gr. dose of sulph. quinine, some hours before the attack, to cure an intermittent fever. A free dose of morphine procured sleep, after which the patient awoke and continued in his right mind.

Art. 2.—*A case of Strangulated Hernia in a man aged* 82 *years.* By Dr. J. Harvey, of Ohio.—Hernia, at the advanced age of 82 years successfully operated upon, from the nature of things must be a rare occurrence. The suggestion of applying ice previous to an operation, to prevent hemorrhage, is a good one, and may be applicable in other cases than the one here tried.

Art. 3.—*Case of Placenta Previa.* By W. Butterfield, of Ottawa.—This is a case showing that the presentation of the placenta does not always occasion violent hemorrhage, even when left to the unaided powers of nature. How far the death of the child, and consequent suspension of the fœtal circulation may have been the cause of absence of hemorrhage, is difficult to say. Generally, in such cases, the flooding will be jointly from the matrix and the placenta.

Art. 4.—*Case of Encephaloid Tumor.* By John Cook, M. D., of Vt.—This is but another of the many melancholy proofs of the malignancy of this disease. The removal of a tumor of this class appears to afford but temporary exemption, as it soon manifests itself in the same, or some other, and generally a more vital part of the system. No doubt remains of the disease being constitutional and the tumor a local manifestation of it.

Art. 5.—*Case of Hydrocephalus Internus successfully treated by Iodide of Potassium.* By L. Brackett, M.D., of Ind.—This case is well reported, the symptoms being given, which adds greatly to the interest of the case, as where they are not the profession must rely upon the knowledge and judgment of the reporter for the diagnosis.

The success of the medicine in this case highly recommends it to the notice of the profession.

No. VI. Art. 1.—*On the Treatment of Fevers and Inflammations without Mercury.* By A. G. Henry, M.D., of Pekin.—We have long concurred in the sentiment of the author that most cases of fevers might be successfully treated without the use of mercury. We would however observe the admission that in many cases benefit may be obtained from it—where the liver is torpid it is too well demonstrated that mercury or its preparations are indicated for them to be abandoned readily.

The suggestion of using as a substitute for mercury from four to six grain doses of opium, is as far as we know original with Dr. H., and if the practice is as successful in other hands as it has been in his, will prove of great value.

We hope we will be favored with more frequent communications from Dr. H. in future.

Art. 2.—*A Case of Idiopathic Tetanus.* By W. Wadsworth, M.D., of Wisconsin.—Tetanus both idiopathic and symptomatic is fortunately a comparatively rare disease.

Art. 3.—*On the Use of Quinine.* By A. W. Benton, of Sterling.—The author thinks that quinine acts by neutralizing "the sedative poison of malaria" in the cure of fever.

Art. 4.—*Case of Abscess of Female Breast from which a worm was discharged.* Reported by Prof. Brainard.—This case is unique, and it would be interesting to learn from Dr. Mead the result of the second abscess.

Art. 5.—*Reduction of Dislocation of the elbow of five months standing.* By Prof. Brainard.—This is an interesting case, and we shall hope at some future period to be able to give the final result of the treatment.

Art. 6.—*On the use of Nitric Acid Issues in chronic diseases.* By P. A. Allaire, M.D.—The experience of the author and the laws of chemical reaction point out nitric acid as a most excellent agent for the establishment of issues. The speed of its action and the profuse suppuration it induces, must highly recommend it. The author is much in favor of large issues.

Art. 6.—*Letter from Dr. Herrick.*—This gives an interesting account of the manners and customs of the Mexicans, and of the diseases and their treatment in our army.

From this brief recapitulation it is seen that our friends have most liberally contributed to our pages; and in reference to this we would observe, that contributions to the pages of a medical journal form the surest evidence that a physician is striving to keep pace with the science, and the plainest road to professional distinction. Few men will thoroughly investigate a subject, unless they are about to commit the result of their investigation to writing. The habit of investigating and writing may stand to each other in the relation of cause and effect, and may operate alternately. When one writes he should investigate thoroughly; and when he investigates thoroughly he is almost certain to write.

*Reviews.*—Our Review Department has been filled with reviews of the most important of the new publications as they have been issued from the press. We feel confident that our labors in this respect have been satisfactory and instructive to our readers.

*Bibliography.*—We have endeavored to notice all works, pamphlets, books, &c., which have been sent to us, and if we have made any omission in this respect, we ask that it may be attributed to oversight and not to intention.

Of the editorial matter we will not speak—but if the reader has any curiosity to refresh his memory in reference to it, he may turn back to that head in each number.

*Abstracts.*—This head will be found to be very interesting in several of our numbers. The researches in reference to the physiology and pathology of the blood, in numbers one and two, will be found to be exceedingly interesting.

*Selections.*—In making selections we have been governed to a great extent, by the consideration of usefulness in practice, hence our articles will many of them be found to be practical; yet we endeavor to glean from our cotemporaries all that our space will allow that is new, practical, curious, and otherwise interesting. E.

